# 

**Published:** 1924-01

**Authors:** 


					t?; 11
New Series. Vol. III. No. 28 (vjk? )? January, 1924. Monthly, Price 6d.4POS7T?E)
m m

				

